# Oral health related quality of life and determinant factors in patients with head and neck cancer

**DOI:** 10.4317/medoral.22670

**Published:** 2019-04-24

**Authors:** Niebla-Bezerra de Melo, Válery-Muniz de Sousa, Ítalo-de Macedo Bernardino, Daniela-Pita de Melo, Daliana-Queiroga-Castro Gomes, Patrícia-Meira Bento

**Affiliations:** 1 Msc. Department of Dentistry, State University of Paraíba, Campina Grande, Brazil; 2 Department of Dentistry, State University of Paraíba, Campina Grande, Brazil; 3 PhD. Department of Dentistry, State University of Paraíba, Campina Grande, Brazil

## Abstract

**Background:**

The present study aimed to measure the impact of oral health on the quality of life of patients with head and neck cancer.

**Material and Methods:**

A cross-sectional study was conducted with 130 patients diagnosed with head and neck cancer at two medical centers. Participants answered a sociodemographic questionnaire and the Oral Health Impact Profile - 14 (OHIP-14). Clinical aspects, cancer staging, and treatment approach were also investigated. Mann-Whitney and Kruskal-Wallis non-parametric tests were used for statistical analysis, followed by Poisson regression analysis (with robust error variance), to associate the OHIP-14 scores with independent variables.

**Results:**

The OHIP-14 presented good internal consistency (Cronbach’s Alpha = 0.861). The mean score obtained was 19.52 (±11.79). Physical pain (3.70±2.44), physical disability (3.26±2.62) and functional limitation (3.24±2.45) were ranked as the main factors affecting the quality of life. Patients non-Caucasians (PR = 1.30; IC 95% = 1.07-1.58; *p* = 0.009), widowers (PR = 1.36; IC 95% = 1.13-1.64; *p* = 0.001), diagnosed with squamous cell carcinoma (PR = 1.28; IC 95% = 1.05-1.58; *p* = 0.017) and with temporomandibular pain (PR = 1.31; IC 95% = 1.08-1.60; *p* = 0.007) were more likely to exhibit lower rates of quality of life.

**Conclusions:**

The results showed a high impact of the oral health in the quality of life of patients with head and neck cancer was observed. Sociodemographic and clinical characteristics can exert influence on the quality of life of patients with head and neck cancer.

** Key words:**Quality of life, malignant neoplasms, head and neck neoplasms, oral health, epidemiology.

## Introduction

Head and neck cancer (HNC) is ranked 6th in a global scale – with an annual incidence ranging from 400.000 to 700.000 new cases (1,2). The oral cavity and the larynx are the most affected regions in the head and neck (3,4). In 2012, 300.400 new cases of cancer in the oral cavity and 156.877 new cases of cancer in the larynx were reported in the world (1).

In most of the cases, head and neck tumors may destroy organs that play an important part in daily activities such as eating and speaking (5). Additionally, the therapeutic approaches for the treatment of cancer usually result in collateral effects (6,7). that impact in the quality of life. The assessment of the quality of life in patients with cancer became a valuable tool to investigate the progression of the disease and the effectiveness of the treatment (8).

The oral condition has an essential role in the individual’s systemic health (9). In specific, the quality of life-related to oral health (OHRQoL) may be defined as the lack of negative impact of the oral condition on social, psychological and functional activities (10). In general, patients with tumors in the oral cavity figure amongst the worst indices for the quality of life when compared to patients with tumors in other regions (11). Based on the exposed, the present study aimed to measure the impact of OHRQoL of patients with HNC and to evaluate the factors associated.

## Material and Methods

-Study design and setting

A cross-sectional study was conducted with 130 patients under treatment for HNC at two medical centers belonging to the Unified Health System (SUS) between November 2016 and April 2017. These institutions are located in Northeastern Brazil and are a reference for about 69 municipalities, covering a population of approximately 1,025,343 inhabitants. The study region has significant social, economic, and cultural disparities. Patients were selected consecutively since consecutive sampling is typically better than convenience sampling in controlling sampling bias.

All the procedures performed in the present study were approved by the Committee of Ethics in Research of the State University of Paraiba, under the protocol number: CAAE 61101716.9.0000.5187 in accordance with Resolution 466/12 of the National Health Council (CNS) and the Declaration of Helsinki. All the patients were asked to sign informed consents prior to data collection. The STROBE (STrengthening the Reporting of OBservational studies in Epidemiology) checklist was used to assist in conducting the survey.

-Eligibility criteria

The eligibility criteria were: patients diagnosed with HNC before or during anti-neoplastic treatment (surgery/chemotherapy/radiotherapy); patients over 18 years of age and patients with altered cognitive ability.

-Training and calibration exercise

Prior to data collection, a pilot study and training and calibration procedures were conducted. Twenty patients were selected to answer the questionnaires. After an interval of 7 days, the questionnaires were applied again to determine the agreement of responses. In this step, the participants did not present difficulties to understand the questions and, therefore, the data collection instruments were not modified. Kappa test values ranged from 0.81 to 0.95, indicating excellent concordance.

-Non-clinical data collection

The acquisition of nonclinical data involved the administration of a questionnaire containing sociodemographic and economic variables and the Oral Health Impact Profile (OHIP-14) to evaluate the OHRQoL. Sociodemographic variables were categorized as follows: age (≤ 39 years / 40-49 years / 50-59 years / 60-69 years / 70-79 years ≥ 80 years), sex (male / female), occupation (retired/farmer/other), self-declared skin color (Caucasian / non-caucasian), marital status (married / single / widowed / divorced), monthly income (<1 Brazilian minimum salary / 1- 2 Brazilian minimum salaries / >2 Brazilian minimum salaries).

The OHIP-14 is a validated instrument to assess the impact of oral problems in the quality of life composed of 14 questions that measure individual perception about the biopsychosocial impact of oral disorders associated with quality of life, providing a comprehensive detection of discomfort, disability, and dysfunction attributed to poor oral conditions. High scores indicate a greater impact of oral health on quality of life and, consequently, worse OHRQoL (12-14).

-Clinical data collection

The clinical data collection was conducted in the medical records of each patient. Data were extracted from patient charts and recorded on a specific individual clinical chart for the study addressing anatomic location of the lesion (oral cavity / pharynx / larynx / neck region / other), diagnosis (squamous-cell carcinoma / Metastatic carcinoma / Adenocarcinoma / Non-Hodgkin lymphoma / Hodgkin lymphoma / Osteosarcoma / other), clinical staging (initial – I or II / advanced – III or IV), treatment stage (before / during); chemotherapy (yes / no); radiotherapy (yes / no); last visit to the dentist (< 6 months / 1-2 years / > 2 years); pain in the TMJ (yes / no); smoker (yes / no); former smoker (yes / no); alcoholic (yes / no); former alcoholic (yes / no).

-Data analysis

Initially, descriptive statistics were performed to screen the general sample characteristics. The qualitative variables were quantified in absolute and relative frequencies, while the quantitative variables underwent the quantification of central tendency and variability. Mann-Whitney and Kruskal-Wallis non-parametric tests were used to compare the OHIP-14 scores according to the characteristics of the patients. These tests were performed because data normality and homogeneity were not confirmed after Kolmogorov-Smirnov and Levene’s tests, respectively.

Next, Poisson regression analysis (with robust error variance) was performed to associate the dependent variable (OHIP-14 score) with the independent variables (social, demographic and financial information, clinical condition, prognosis, and treatment approach). Variables with a *p*-value <0.25 in the univariate analysis were included in the multivariate model. However, in the final model only the variables with a *p*-value <0.05 were maintained. The prevalence ratio (PR) was calculated for the univariate analysis, while an adjusted PR was calculated for the multivariate analysis (15,16). All the statistical analyses were performed with SPSS 20.0 software (IBM Corp., Armonk, NY, USA) with statistical significance set at 5% (*p*<0.05).

## Results

 Table 1,  Table 1 continue,  Table 1 continue-1, expresses the sample distribution based on social, demographic, financial characteristics, the clinical condition of the patient, the prognosis and the treatment approach of the patients. Most of the patients were males (n = 91; 70.0%) aged between 60 and 69 years old (n = 36; 27.7%) with monthly income between 1 and 2 minimum salaries (n = 92; 70.8%). Most of the patients had cancer in the oral cavity (23.8%) or larynx (23.8%). Most of the cancer lesions were diagnosed as squamous cell carcinoma (66.2%) in advanced stage (72.1%) i.e., in stage III or IV of the TNM classification of malignant tumors. Reports of pain in the teeth and in the temporomandibular joint reached 16.9% and 36.2%, respectively. Former smokers and alcoholics accounted for 65.4% and 67.7% of the patients.

**Table 1:**
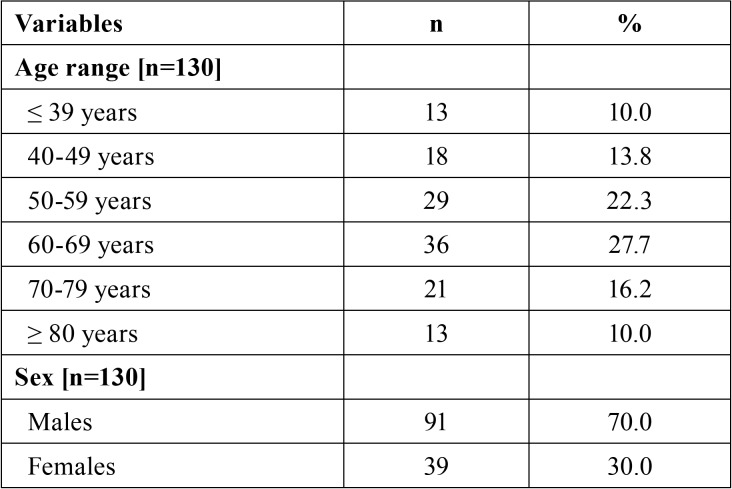
Sample distribution based on social, demographic, financial conditions clinical condition, prognosis and treatment approach.

**Table 1 continue:**
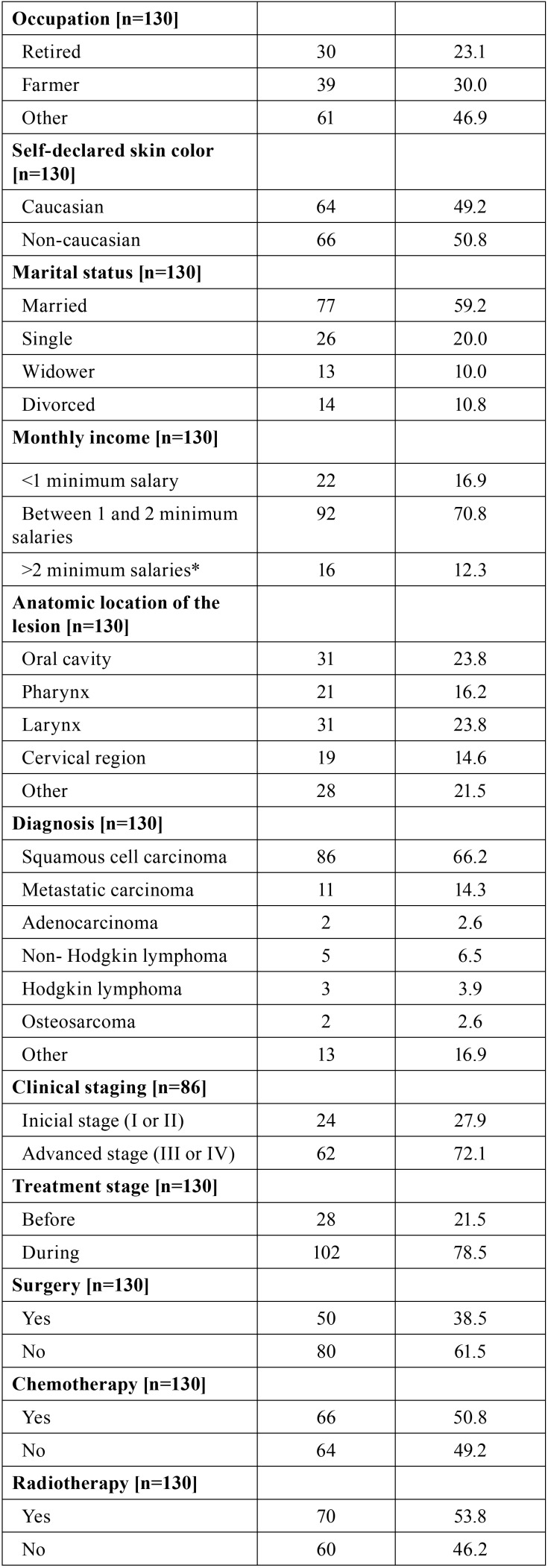
Sample distribution based on social, demographic, financial conditions clinical condition, prognosis and treatment approach.

**Table 1 continue-1:**
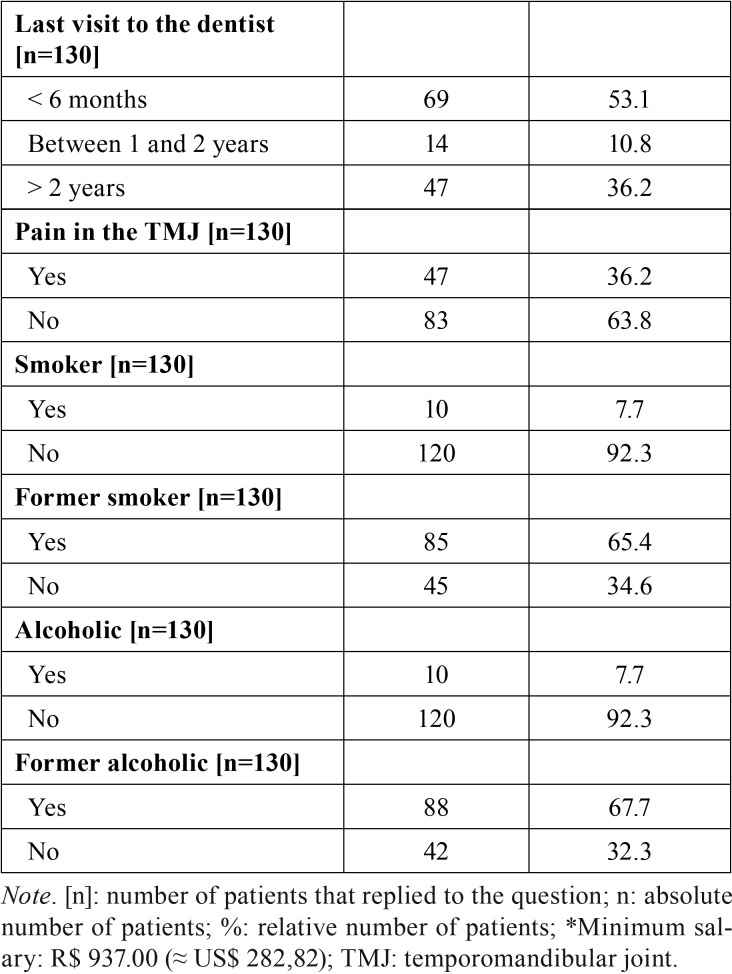
Sample distribution based on social, demographic, financial conditions clinical condition, prognosis and treatment approach.

OHIP-14 had good internal consistency (Cronbach’s Alpha = 0.861).  Table 2 shows the quantification of central tendency and variability of the OHIP-14 scores. The mean score was 19.52 (±11.79). Pain (3.70±2.44), physical disability (3.26±2.62) and functional limitation (3.24±2.45) were ranked as the main factors affecting the quality of life.

**Table 2:**
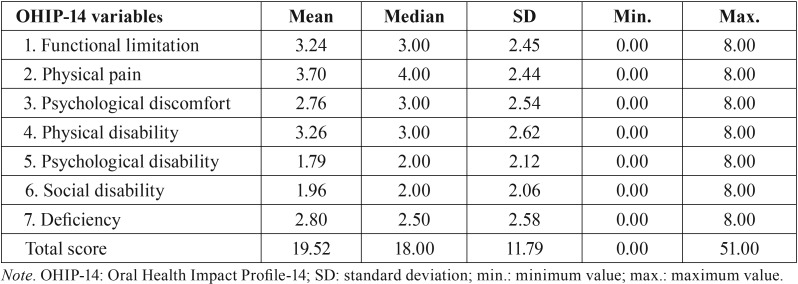
Measurements of central tendency and variability of the scores obtained with the OHIP-14 index.

 Table 3,  Table 3 continue,  Table 3 continue-1 expresses the outcomes of the comparative analysis of OHIP-14 scores based on the clinical condition of the patient, the prognosis and the treatment approach. Differences statistically significant were observed for the following variables: self-declared skin color (*p* < 0.05), anatomic location of the lesion (*p* < 0.05), dental pain (*p* < 0.05) pain in the temporomandibular joint (*p* < 0.05).

**Table 3:**
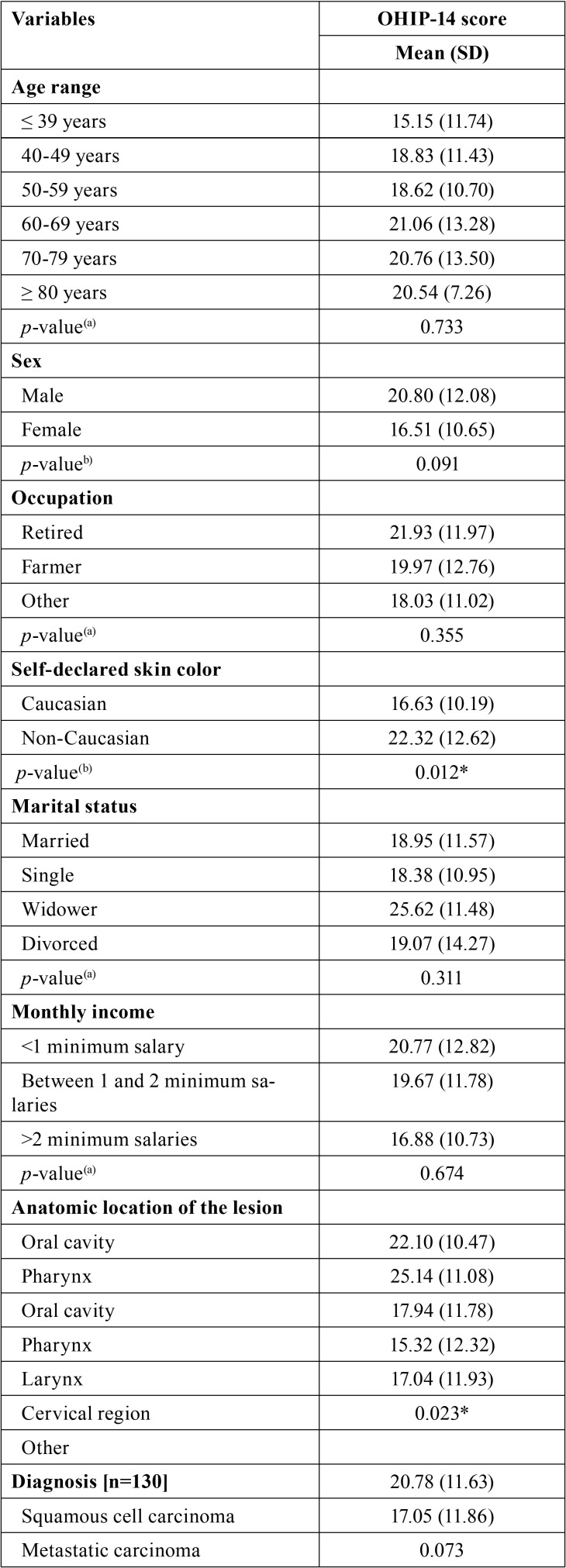
Comparative analysis between the OHIP-14 scores and the social, demographic, financial and clinical conditions, as well prognosis and treatment approach.

**Table 3 continue:**
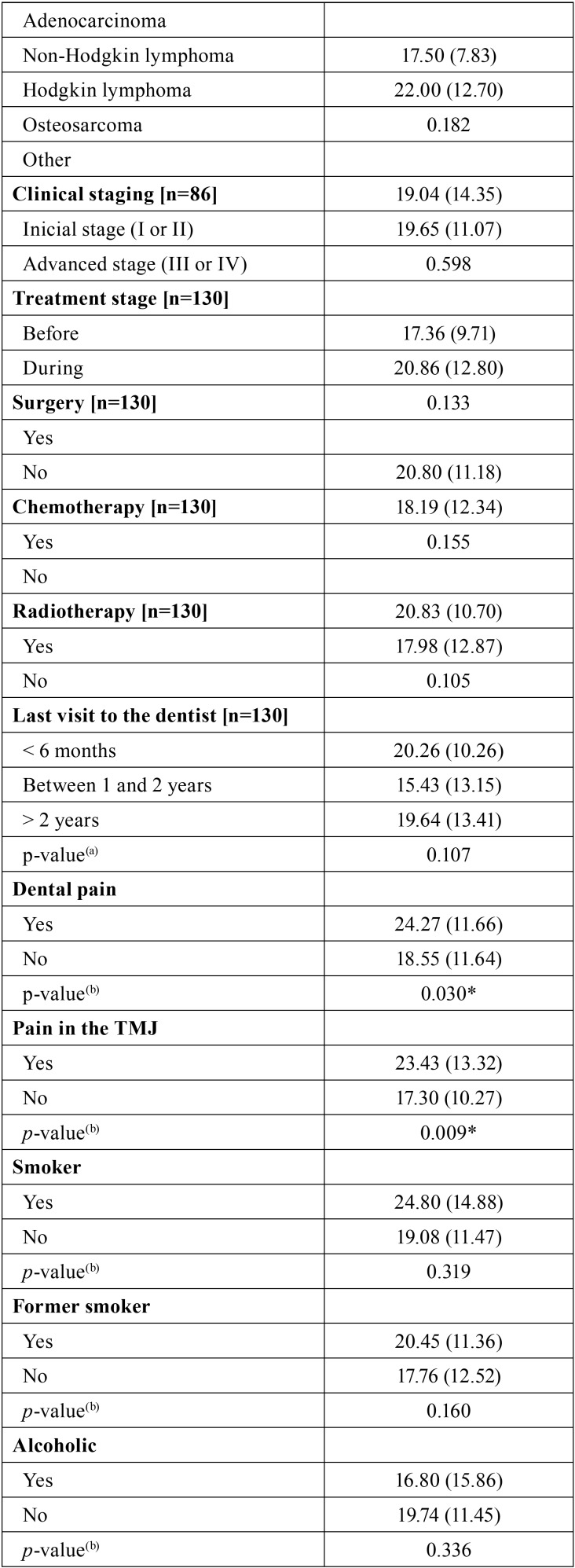
Comparative analysis between the OHIP-14 scores and the social, demographic, financial and clinical conditions, as well prognosis and treatment approach.

**Table 3 continue-1:**
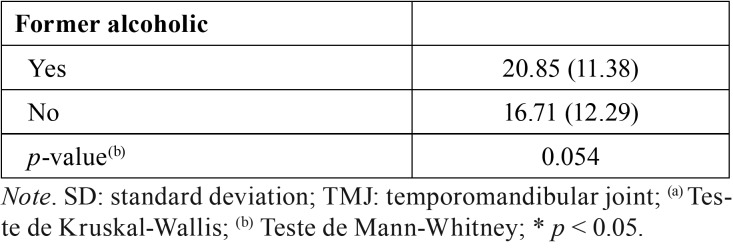
Comparative analysis between the OHIP-14 scores and the social, demographic, financial and clinical conditions, as well prognosis and treatment approach.

 Table 4,  Table 4 continue expresses the outcomes of Poisson regression analysis based on the OHIP-14 total scores and the variables investigated. Considering the final multivariate model, the factors associated with the decrease in the quality of life were the self-declared skin color (*p* < 0.05), marital status (*p* < 0.05), diagnosis of the lesion (*p*p < 0.05) and pain in the temporomandibular joint (*p* < 0.05). Non-Caucasians (PR = 1.30; IC 95% = 1.07-1.58; *p* = 0.009), widowers (PR = 1.36; IC 95% = 1.13-1.64; *p* = 0.001), patients diagnosed with squamous cell carcinoma (PR = 1.28; IC 95% = 1.05-1.58; *p* = 0.017) and patients with pain the temporomandibular joint (PR = 1.31; IC 95% = 1.08-1.60; *p* = 0.007) had more propensity to reach higher scores in the OHIP-14 – indicating potential impact in the quality of life.

**Table 4:**
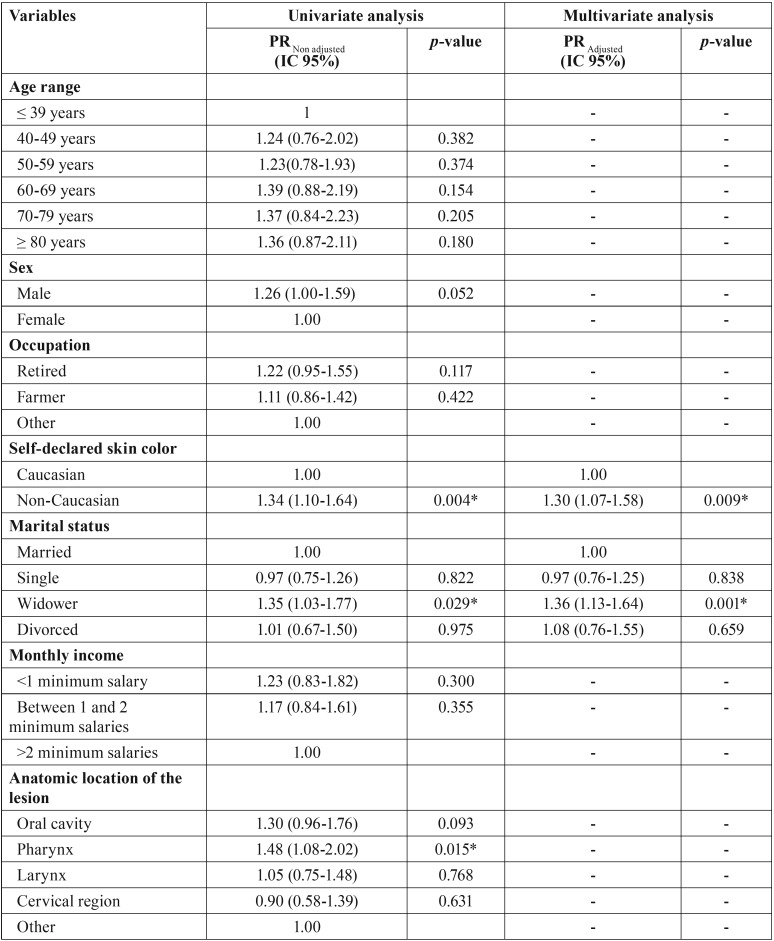
Poisson regression analysis for the OHIP-14 score based on the social, demographic, financial and clinical conditions, as well prognosis and treatment approach.

**Table 4 continue:**
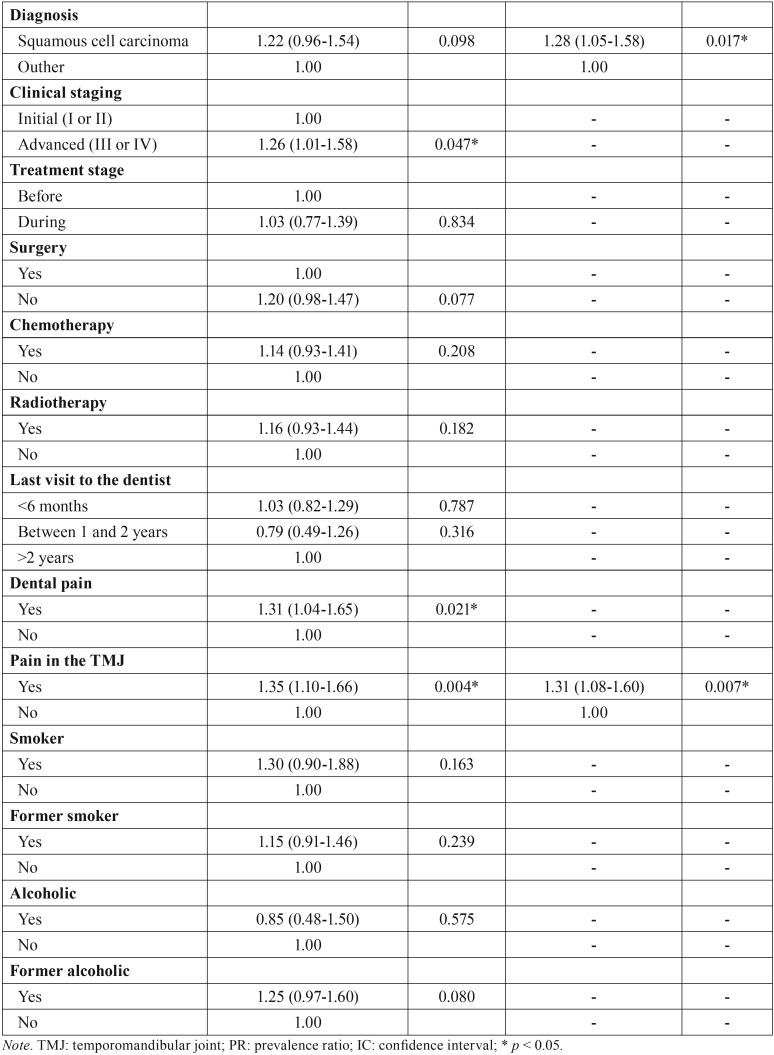
Poisson regression analysis for the OHIP-14 score based on the social, demographic, financial and clinical conditions, as well prognosis and treatment approach.

## Discussion

Malignant neoplasms of the head and neck affect more often male subjects aged above 40 years of age. Nearly 90% of these tumors are diagnosed as squamous cell carcinoma. In most of the cases, the malignant lesions are detected in an advanced stage (stages III and IV) (17-19) in the oral cavity, the larynx and the pharynx (18,20). These findings were also observed in the sample investigated in the present study. Furthermore, the found prevalence of former smokers and alcoholics, as well as patients that work in the countryside highlights some of the risk factors for HNC (19-21).

In relation to the oral health habits, the patients of the present study reported the previous consultation with a dentist before the antineoplastic treatment. This finding may be justified by the dental services offered by both hospitals visited during the study. However, it is important to note that a large part of the sample reported the previous consultation with a dentist dated more than 2 years ago.

In the present study, the OHIP-14 reached good internal consistency. The OHIP-14 pointed towards a high impact in the quality of life of the patients sampled. Patients with HNC experience reported a statistically superior impact on quality of life when compared to cancer-free individuals (22). In the present study, the main complaints reported by the patients were the physical pain, physical disability, and functional limitation. These outcomes corroborate with Barrios *et al.* (2015) (23) and Stuani *et al.* (2018) (22). Head and neck tumors can destroy the integrity of surrounding organs that are crucial to essential human functions leading to profound physical changes (24).

Patients self-declared non-Caucasians, widowers and diagnosed with squamous cell carcinoma and with pain in the temporomandibular joint had more propensity to reach higher scores in the OHIP-14 in this study. There is no consensus in the scientific literature on the influence of skin color in the quality of life, however, this result can be explained by socioeconomic factors (25,26). Marital support has an essential part in the improvement of the clinical condition in patients with cancer (27). In this context, patients that lost their partner may experiment higher negative impact in their quality of life – especially when affected by a disease. Additionally, patients diagnosed with squamous cell carcinoma expressed through the OHIP-14 scale a high impact in their quality of life. It highlights and confirms a decrease in their quality of life-related to oral health (28). In relation to the temporomandibular joint, the local expansion of the head and neck tumor itself (or its metastasis) together with the antineoplastic treatment may damage adjacent structures in the maxillofacial complex, such as muscles, neural bundles, supporting tissues and the temporomandibular joint (29). Consequently, the complaint of pain is reported expressing a negative impact on the quality of life.

Measuring the quality of life of patients in relation to their health condition became more important over time (18). This procedure may be used as an indicator of the effectiveness of the treatment and the specific areas in which the patient needs more attention (30). For that reason, the patient must be examined in total with special attention to oral health, which has an important role in the quality of life of patients with HNC (10).

This study has some limitations. Due to the cross-sectional design, it is not possible to establish causal relations. In addition, responses in the questionnaires may have been subject to information bias. However, a number of measures were taken to lessen the occurrence of such bias, such as using a validated questionnaire and conducting a pilot study. Besides that, a wide variety of histological types of tumors were observed in the study. However, the majority of the sample had tumors in similar clinical staging (advanced stage), allowing the comparison between them.

On the other hand, the present study contributed to the advancement of scientific knowledge for three main reasons. First, it evaluated the OHRQoL in patients with head and neck cancer. Second, it allowed identifying determinants factors of OHRQoL. Third, the results of this study may help in the implementation of public health policies.

## Conclusions

A high impact on the quality of life-related to oral health was observed in the present study. Self-declared skin color, marital status, diagnosis and pain in the TMJ were factors associated with the OHRQoL. More specific patients self-declared non-Caucasians, widowers, and those diagnosed with squamous cell carcinoma and temporomandibular pain hade more propensity for higher scores in the OHIP-14 – indicating potential impact in the quality of life.
